# Multitemporal Land Use and Cover Analysis Coupled with Climatic Change Scenarios to Protect the Endangered Taxon *Asphodelus bento-rainhae* subsp. *bento-rainhae*

**DOI:** 10.3390/plants12162914

**Published:** 2023-08-10

**Authors:** Alice Maria Almeida, Fernanda Delgado, Natália Roque, Maria Margarida Ribeiro, Paulo Fernandez

**Affiliations:** 1 School of Agriculture, Polytechnic University, IPCB—Polytechnic Institute of Castelo Branco, 6001-909 Castelo Branco, Portugal; 2 CERNAS—Research Center for Natural Resources, Environment and Society, Polytechnic Institute of Castelo Branco, 6000-084 Castelo Branco, Portugal; 3 CEF—Forest Research Centre, Superior Institute of Agronomy, Lisbon University, 1349-017 Lisbon, Portugal; 4 MED&CHANGE—Mediterranean Institute for Agriculture, Environment and Development & CHANGE–Global Change and Sustainability Institute, Évora University, 7006-554 Évora, Portugal

**Keywords:** *Asphodelus bento-rainhae* subsp. *bento-rainhae*, climate change, conservation, species distribution modeling, MaxEnt, LULC

## Abstract

Climate change and land use and land cover (LULC) change are impacting the species’ geographic distribution, causing range shifts and reducing suitable habitats. *Asphodelus bento-rainhae* subsp. *bento-rainhae* (AbR) is an endangered endemic plant restricted to Serra da Gardunha (Portugal), and knowledge of those changes will help to design conservation measures. MaxEnt was used to model AbR’s current distribution and project it into the future, 2050, using the Shared Socioeconomic Pathway SSP3-7. The Portuguese LULC maps from 1951–1980, 1995, 2007, and 2018 were used to assess and quantify LULC changes over time. The results showed that the AbR current predicted distribution matches its actual known distribution, which will not be affected by future predicted climate change. The significant LULC changes were observed during the study periods 1951–1980 to 2018, particularly between 1951–1980 and 1995. Scrubland and Agriculture decreased by 5% and 2.5%, respectively, and Forests increased by 4% in the study area. In the occurrence area, Agriculture increased, and Forests decreased between 1980 and 2018, due to Orchard expansion (34%) and declines in Chestnut (16.9%) and Pine (11%) areas, respectively. The use of species distribution models and the LULC change analysis contributed to understanding current and future species distribution. The LULC changes will have a significant impact on future species distribution. To prevent the extinction of this endemic species in the future, it is crucial to implement conservation measures, namely species monitoring, replantation, and germplasm conservation, in addition to guidelines for habitat conservation.

## 1. Introduction

Climate change will lead to an increase in temperature and drought, which, together with floods and forest fires, will raise considerable challenges for the Mediterranean region in the future, with direct consequences for species existence and distribution. The climate models clearly show that the Mediterranean region is particularly prone to global warming this century [1,2]. Precipitation will decrease as temperatures rise; the latter will show a 20% increase compared to the world global average in the last decade. The summer warming will be dramatic, possibly doubled, compared to worldwide global change [1]. Global change may drive changes in the species distribution area, leading to geographical ranges shifts in the future [3]. The species might respond by short-term acclimation, long-term adaptation, and altitudinal and latitudinal migrations in future climate [4] due to habitat loss and climatic conditions changes in their range-wide distribution [2]. Possible expansion of arid areas in a fragmented landscape may exacerbate the global warming effects on the species of the Mediterranean region, particularly the ones already in danger [2], and projected future precipitation changes together with increasing temperatures will have dramatic consequences in terms of increased drought and fire events’ intensity/frequency [5]. In the Mediterranean region, Portugal is the world’s 36th biodiversity hotspot due to the large number of rare plant species present in this region, and considering the serious threats posed by habitat alteration and climate change, those species ought to be studied [6]. In this paper, *Asphodelus bento-rainhae* P. Silva (Asphodelaceae), an endemic species of the Iberian Peninsula, known in this region by abrótea, abrótega, gamão or bengala-de-são-josé, and first described in 1956 by the botanist and agronomist Pinto da Silva [7], was studied. The genus *Asphodelus* is a circum-Mediterranean genus, which includes five sections and 18 species [8].

According to Raunkiær (1934) [9] and other authors [7,10,11], this species is a rhizomatous geophyte with tuberous-fasciculate roots, linear and glaucous leaves, and inflorescences (single or compound bunch) supported by a smooth floral stem and brownish-black floral bracts; the flowers have oblanceolate white tepals with a brownish-red vein and longer stamens, and the fruit is a mitriform capsule, with inconspicuous veins before maturation and transversely rough-veined in dehiscence, with obcordate, oblong or oblong-ovate valves where the seeds are sharply trigonal, attenuated in the extremities and black.

Díaz-Lífante and Valdés (1996) [11] consider the existence of two subspecies, *A. bento-rainhae* P. Silva subsp. *bento-rainhae* (AbR) (Serra da Gardunha, Portugal) and *A. bento-rainhae* P. Silva subsp. *salmanticus* Z. Díaz & Valdés (Provinces of Ávila, Salamanca and Cáceres, Spain). The habitats of the first subspecies are the understories of Galicio-Portuguese oak woods (9230—Rede Natura 2000, with *Quercus robur* L. *Quercus pyrenaica* Willd.), *Castanea sativa* Mill. woods (9260), and *Prunus avium* L. orchards, those mainly on path slopes and edges without herbicide application and frequent soil mobilization [12]. According to Travassos (1999) [13] and Delgado (2010) [14], the particular tree canopy that existed until the middle of the 20th century may have influenced the adaption and evolution of AbR to those particular habitats. Additionally, AbR may also be found in mosaics with other shrub communities or with living trees, like those in the class *Stipo-Agrostietea castellanae* that belongs to the alliance *Agrostion castellanae*. This class has particular vegetation as *Centaurea aristata* and *Dactylis glomerata* subsp. *lusitanica*, and *Rumex acetosella* subsp. *angiocarpus*, in certain soil types, such as deep granites and schist-greywacke. AbR continues to exist on orchard path slopes, although many of the *Stipo-Agrostietea castellanae* species were not confirmed there [15]. AbR is prevalent in the western zone of Serra da Gardunha, with a more temperate and humid environment than the eastern zone, which is colder and dryer [16]. AbR is a conservation-prone species, for it is endemic and only exists in a narrow area (roughly 7 km^2^, Serra da Gardunha, Portugal), has fragmented population structure, a very low effective population size (number of breeding individuals), and a low number of individuals. Additionally, it is only found on specific Serra da Gardunha slopes (a central Iberian mountainous belt), at elevations from 530 to 940 m [11,17].

The Western Mediterranean is the center of diversification of the genus since most of the species included are exclusive to this region, and in *Asphodelus*, the processes that lead to speciation are, principally, polyploidy, reproductive isolation and hybridization, which affect the species of the genus differently, since seed germination and seedling development are of great taxonomic importance [11]. Sexual reproduction is present in all taxa of the genus *Asphodelus*, but the incidence of this type of reproduction each year and in each population varies according to the joint existence of a vegetative multiplication, mainly in perennial species with a long life cycle [11] such as AbR. They find in the genus species that can be classified between facultative autogamous and obligatory autogamous. *A. tenuifolium* Cav. and *A. fistulosus* L. are among the group of facultative allogamous species, and the rest are between these and the obligatory allogamous [11]. In a reproductive biology study with *A. aestivus* Brot., cross-fertilization by insect pollination is required for seed production due to the absence of selfing [18]. Moreover, in the referred species, the breeding system indicates that sexual reproduction is relatively inefficient, and active vegetative propagation exists; a similar situation is likely found in AbR. Indeed, the low AbR gene flow is influenced by very short-distance seed dispersal and pollen dispersed by insects.

Furthermore, the seed’s hypogeal germination and dormancy also induce low germination rates. Together, clonal propagations and low seed dispersal might lead to strong endogamy and biparental inbreeding, which increase homozygosity and diminish species genetic variability. Indeed, this species exhibits little intraspecific genetic diversity (2%) [19], which confirms the asexual reproduction preference and putative inbreeding [17], leading to genetic drift as a putative driver in this taxon’s speciation [11] and the location of AbR in a very circumscribed place (Serra da Gardunha).

Some studies verified that the dormancy mechanisms affect germination [20,21]; this species exhibits physical dormancy, which can be alleviated by using various treatments [22,23]. According to Delgado (2010) [14], the tegumentary dormancy of the seeds was broken through mechanical cutting, allowing for 84% germination in fresh seeds and 58% germination in seeds stored for 2 years. Temperatures of 15 °C and a photoperiod of 8 h of light indicate that germination will occur in nature during the autumn season. Furthermore, because it is a geophytic plant with a fire-resistant underground root structure and a tendency to fill in places that have recently burnt and lack a tree canopy, this species is thought to be a sign of environmental degradation [19].

AbR might have economic value. A recent study characterized the substances in the dried root tubers and showed that terpenoids were the main class of secondary metabolites in the extracts [24]. The leaves of AbR were used as food and in traditional medicine to treat ulcers and urinary and inflammatory disorders [25], and also as fertilizer and fodder in Portugal [26]. The ethyl ether fractions demonstrated the highest antibacterial activity against all Gram-positive microorganisms, with aloe-emodin as one of the main marker compounds that are highly active against *Staphylococcus epidermidis*, and the ethyl acetate fractions exhibited the highest antioxidant activity [25].

AbR was considered vulnerable on the International Union for the Conservation of Nature (IUCN) Red List of Threatened Species [27] and was assessed as “Endangered” by the Portuguese Red List of Vascular Flora [28] due to the continued decline in the area and quality of its habitat as a result of orchard expansion, the use of herbicides, and the change in and degradation of forest habitats caused by recurrent fires that potentiate the appearance of invasive species, and the reconversion of deciduous woods/oak forests into maritime pine stands. Thus, it is considered urgent to ensure the preservation of the species habitat and to promote agricultural and forestry management compatible with species conservation. Nevertheless, despite AbR being considered an “Endangered species”, it is understudied, particularly in terms of the effects of climate change and land use on its distribution.

Species distribution models (SDMs) are widely used in ecology, evolution, biogeography, and conservation. These models allow researchers to identify the most important environmental predictors for species distribution, predict the probability of species occurrence, and predict the species’ presence or absence. They are an important tool to support the definition of endangered species conservation measures. Indeed, modeling the current distribution and predicting suitable habitats can provide conservation stakeholders with information for species conservation planning.

Currently, many species distribution models are available. They can be distinguished by the type of species data involved, the presence-absence or abundance, and the presence-only data [29]. The presence-absence data include species’ presence and absence locations, while presence-only data correspond to the plant occurrence. MaxEnt, a machine learning algorithm, is a widely used presence-only SDM due to its comparatively better prediction performance [30,31,32]. It is consistently used to study the impact of climate change on species distribution [33], endemic species conservation [34], and land-cover classification [35]. For these reasons, this algorithm was chosen to predict the potential distribution of AbR based on presence-only data obtained in fieldwork, the first study carried out for this species.

Knowing how the species will respond to climate change and how it will be affected by land use change could be key for defining conservation strategies. To improve the species knowledge, the aims of the present study were: (i) to predict the current distribution of AbR; (ii) to project its distribution into the future (2050); (iii) to analyze land use change from 1951–1980 to 2018; and (iv) to identify future challenges for the species and propose conservation measures.

## 2. Results

### 2.1. Variable Importance and Model Accuracy

The variables that relatively contributed the most to explaining the model were the BIO4 (temperature seasonality—%) with 68.1%, followed by BIO7 (temperature annual range—°C) (9.8%), aspect (°), soil type, BIO2 (mean diurnal range—°C), BIO15 (precipitation seasonality—%), and BIO12 (annual precipitation—mm) (Table 1). Considering the permutation importance, the most influential variable was BIO4 with 59.69%, followed by BIO2 with 22.46%. The Jackknife test results showed that the variables that explained the most in isolation were BIO4, with the highest gain, followed by BIO12, BIO2, and, soils. The variable that decreased the gain the most when omitted was BIO4, which appeared to have the most information compared to the others. The results of the Jackknife test also showed that using one variable did not exceed the gain of using all variables, indicating that each variable contributed to the improvement of the model’s predictive accuracy (Table 1).

**Figure 1:**
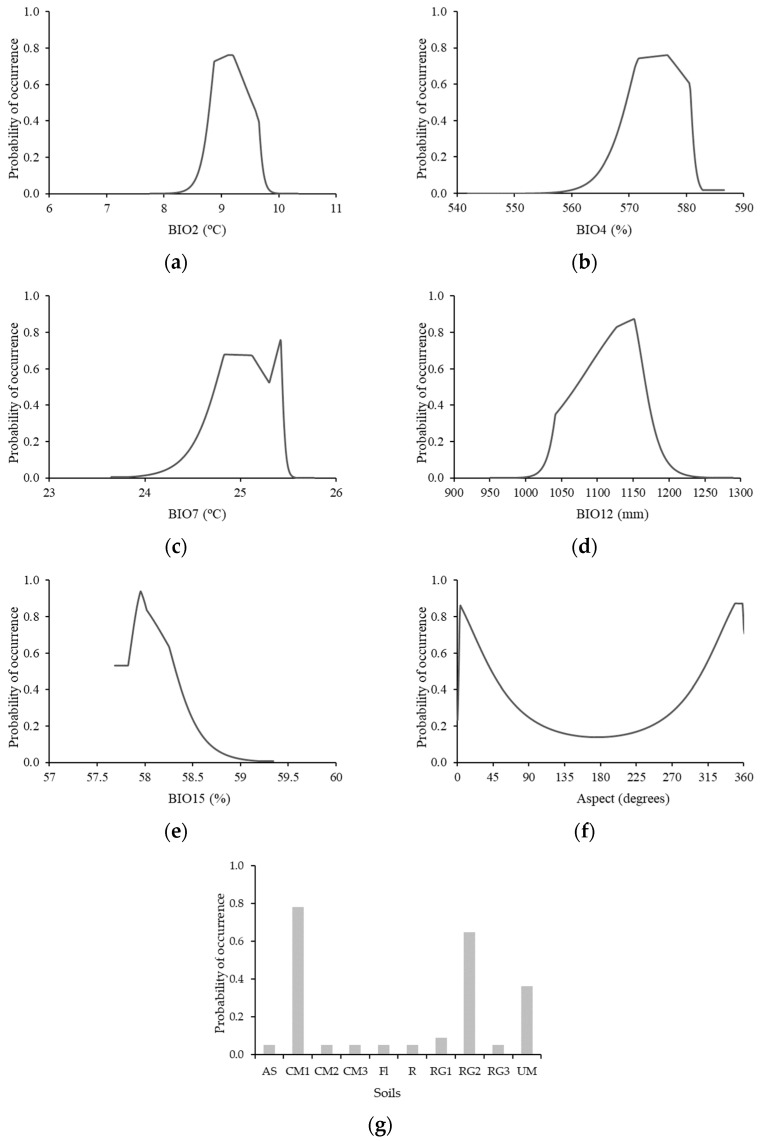
The response curves of predicted suitability values for the selected environmental variables. Mean diurnal range (**a**), Temperature seasonality (**b**), Temperature annual range (**c**), Annual precipitation (**d**), Precipitation seasonality (**e**), Aspect (**f**), and Soil type (**g**). The soil type description is in Table A1.

The response curves (Figure 1) for the environmental variables showed how predicted suitability was affected by each variable independently. In particular, this figure suggests that high suitability (>0.6) is associated with BIO2 between 8.8 and 9.4 °C, BIO4—570–580%, BIO7—24.5–25.5°, BIO12—1080–1160 mm, BIO15—57.8–58.3%, Aspect—2–32°, and 319–360° (N, NE, and NW), and Chromi-Dystric Cambisols (CM1), and Dystri-Epileptic Regosols (RG2) soils (Table A1). The current model was statistically more robust than the random one (AUC = 0.5), with an AUC of 0.91, indicating excellent model precision [31].

### 2.2. Current and Future Potential Distribution

**Figure 2:**
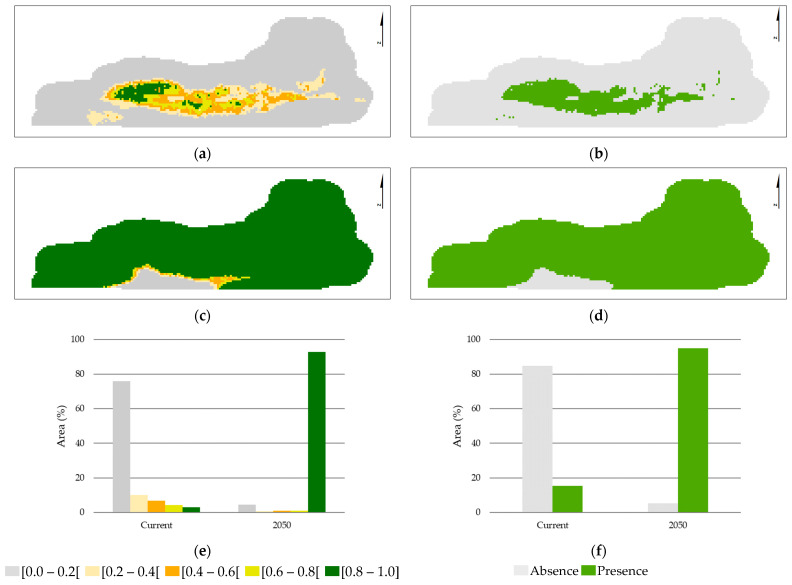
Predicted habitat suitability: Current (**a**) and Future—2050 (**c**). Presence-absence maps: Current (**b**) and Future—2050 (**d**). Suitability areas (%) in the present and for the future (2050) (**e**). Absence/Presence areas (%) (**f**). Suitability class range: [0.0–0.2[, non-suitable area; [0.2–0.4[, low-suitability area; [0.4–0.6[, regular-suitability area; [0.6–0.8[, medium-suitability area; and [0.8–1.0], high-suitability area.

The current model was fitted using the occurrence records and the seven environmental variables previously selected, afterwards used to predict future suitability for 2050 considering the SSP3-7 scenario. The modeling results, predicted habitat suitability, and the presence-absence maps, resulting from applying the 10th percentile threshold (0.364), are presented in Figure 2.

The current and future AbR potential distribution (Figure 2) predicted by MaxEnt revealed that a good correspondence between the distribution area predicted by the model and the known species distribution exists (Figure 2a) and that there will be a considerable species expansion in the future (Figure 2c) as a result of the increase in highly suitable areas [0.8–1.0] (Figure 2e). Those results indicate that environmental conditions will be more suitable for the species’ development than those observed at present. There is a potential expansion of 79.5% in the species’ presence from the present to the future (Figure 2f), suggesting that the suitable area for the species will not be affected by climate change in the coming years (2050); however, it may decrease due to land use change.

### 2.3. Land Use and Land Cover (LULC) Change Analysis

**Figure 3:**
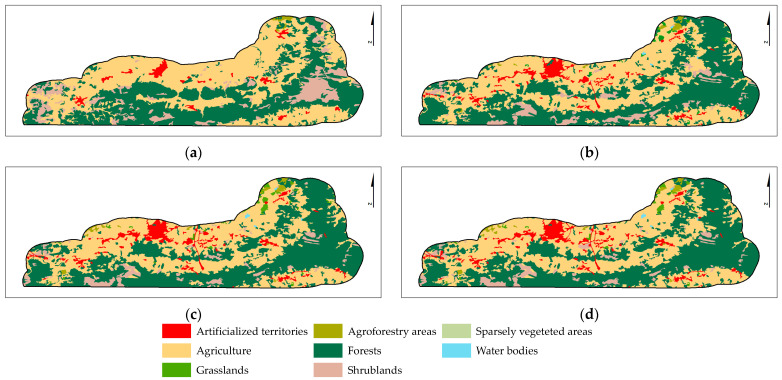
The LULC changes in the study area from 1951–1980 to 2018. MAF1951-80 (**a**), COS1995 (**b**), COS2007 (**c**), and COS2018 (**d**). The LULC class description for the study area (SA) is in Table A2.

The LULC changes in the study area (SA, Table A2) for the overall period (1951–1980 to 2018) are displayed in Figure 3 and Table 2. The results revealed that Agriculture and Forests are the predominant LULC classes in the study area. Agriculture decreased by 2.3%, and Forests increased by 4.1% between 1980 and 2018. The areas occupied by Agriculture and Forests in 2018 represented 44.5% and 43.8% of the study area, respectively. Artificialized territories and the Scrublands also changed significantly between 1980 and 2018. The Scrubland area decreased by 6.5%, and the Artificialized territories area increased by 3.3%.

**Figure 4:**
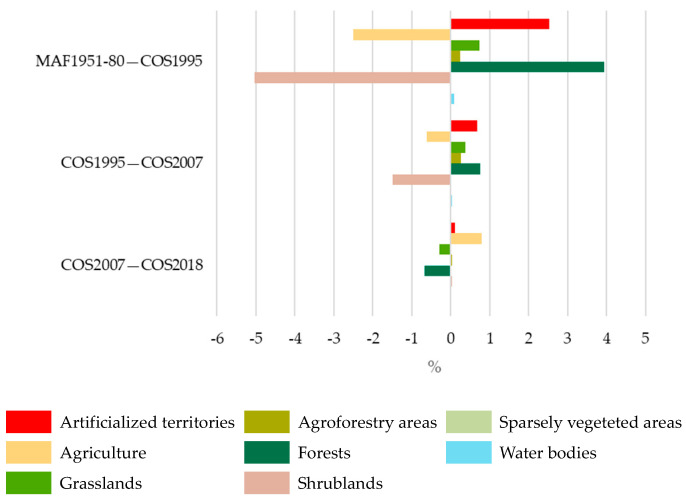
The LULC changes in the study area between two consecutive periods: 1951–1980–1995, 1995–2007, and 2007–2018.

The results also showed that the main changes between two consecutive periods were observed between 1980 and 1995 (Figure 4). Scrubland and Agriculture decreased by 5% and 2.5%, respectively, but an increase of 4% in Forests was verified.

**Figure 5:**
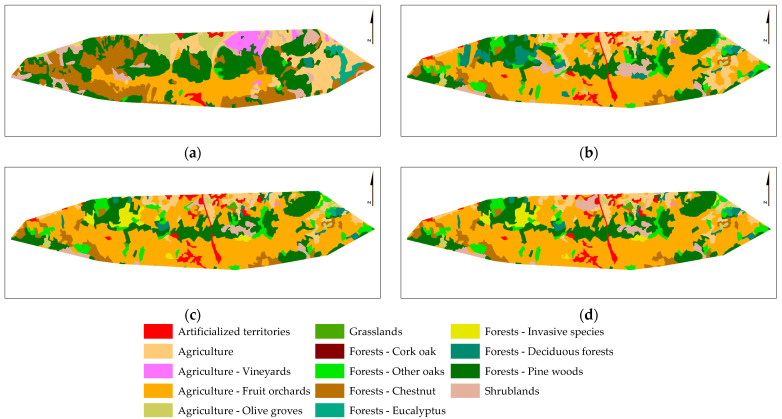
The LULC changes in occurrence area from 1951–1980 to 2018. MAF1951–1980 (**a**), COS1995 (**b**), COS2007 (**c**), and COS2018 (**d**). The LULC class description for the occurrence area (OA) is in Table A2.

**Figure 6:**
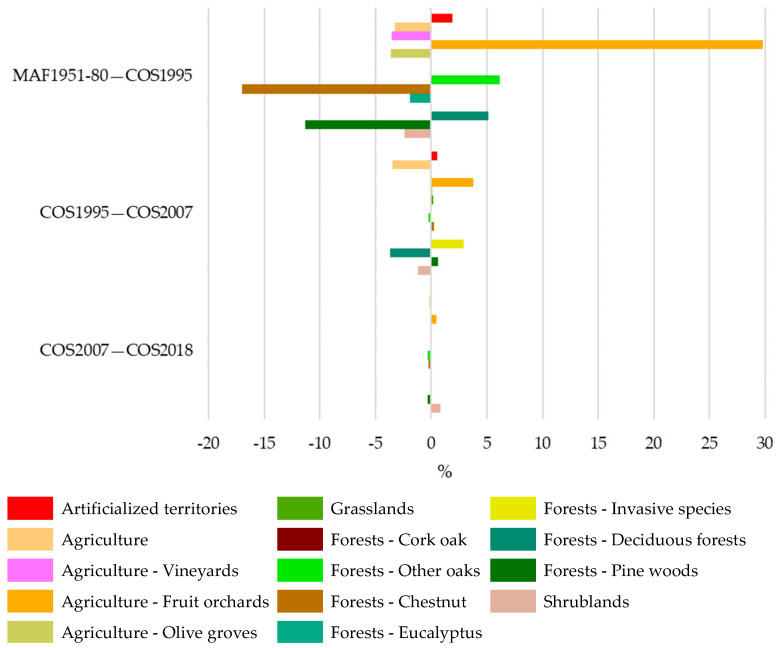
The LULC changes between two consecutive periods: 1951–1980–1995, 1995–2007, and 2007–2018, in the species’ occurrence area.

The analysis of LULC changes in the species occurrence area (OA, Table A2) for the overall period 1951–1980 to 2018 (Figure 5 and Table 3) showed that Agriculture (Agriculture and Permanent crops) and Forest areas predominate, as has been observed for the study area. However, Agriculture increased, and Forests decreased between 1980 and 2018 (Figure 6).

**Figure 7:**
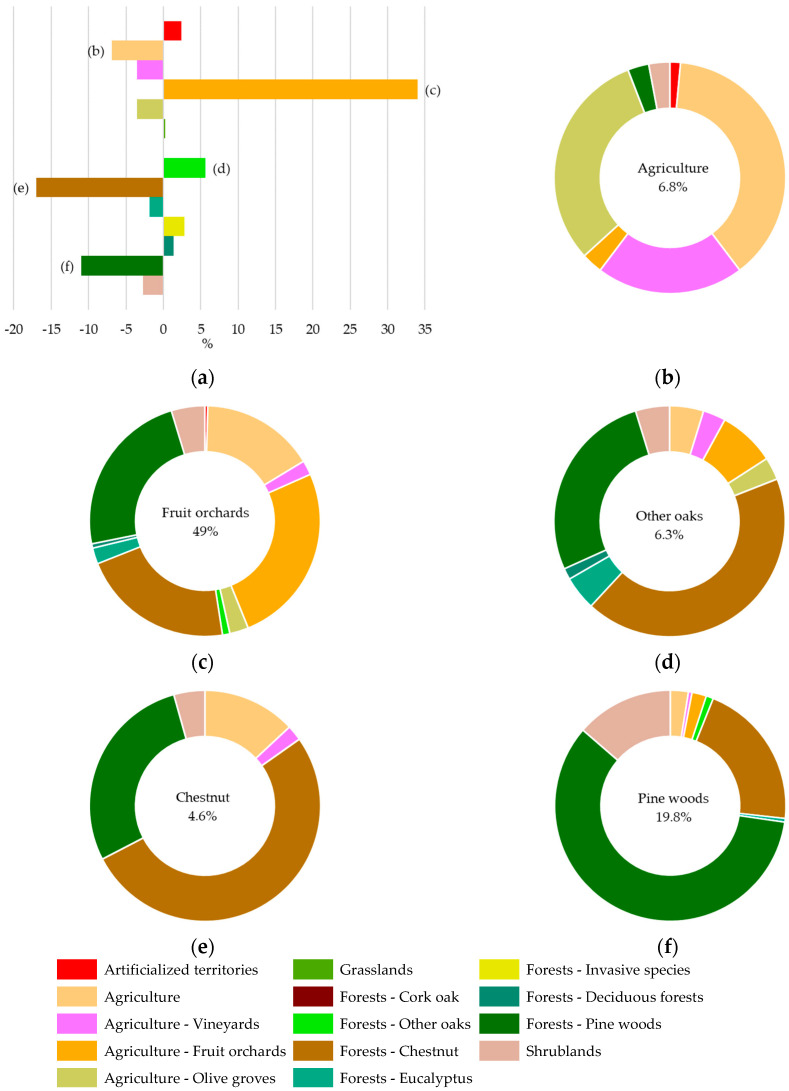
The LULC changes between 1951–1980 and 2018 in species occurrence area (**a**). The LULC inter-class transitions: Agriculture (**b**); Fruit orchards (**c**); Other oaks (**d**); Chestnut (**e**); and Pine woods (**f**).

Figure 7a revealed that there was mainly an increase in Orchards (34%) and Other Oaks (5.7%), followed by Invasive species (2.9%) and Artificialized territories (2.4%) classes, and a reduction in Chestnut (16.9%), Pine woods (11%), and Agriculture classes (6.9%). The reduction in Chestnut, Pine trees, and Eucalyptus areas explains the decline in Forest areas.

Considering all the LULC changes between 1980 and 2018, 30.9% of the LULC persisted for the occurrence area, while 69.1% showed inter-class transitions. The increase in Orchards in 2018 was mainly a result of the transition areas from Agriculture, Pine woods, and Chestnut classes (Figure 7c). The reduction in the Chestnut class was mainly due to the transfer to Orchards, Pine woods, and Other oaks (Figure 7c,d,f).

## 3. Discussion

The MaxEnt model was used to predict the AbR potential distribution under current and future climatic conditions. Considering the AUC (AUC = 0.91), the model had good performance in predicting the AbR suitable habitat. The results suggest that the species distribution was mainly determined by the temperature seasonality (BIO4), the temperature annual range (BIO7), and the aspect, adding up to 85.1% of the model’s total contributions of the environmental variables. Considering the percent contribution, the permutation importance, and the Jackknife test of variable importance, the temperature seasonality (BIO4) was the variable that provided the most information to the model.

The species distribution predicted for the present is in agreement with the known distribution of the species [28,36]. These results are in concordance with those obtained in 2015 by Quinta-Nova et al. [37]. The authors used the AHP multicriteria analysis to map the *Asphodelus bento-rainhae* habitat suitability in Serra da Gardunha Regional Protected Landscape, using bioclimatic, topographic, and soil variables. They concluded that the area most favorable to species occurrence was located on the northern side of the Serra da Gardunha Regional Protected Landscape, coinciding with the current presence of the species. Indeed, the species has a very restricted distribution, occupying a small area on the northern side of the Serra da Gardunha, and is found preferentially on the edges of oak and chestnut forests and the slopes of cherry orchards.

The impact of climate change on the AbR distribution was evaluated for the year 2050. The results showed that climate change considering the Shared Socioeconomic Pathways SSP3-7 scenario, a medium-high reference scenario within the socioeconomic family [38], will not affect the range of the species. On the contrary, the climatic conditions of this scenario for 2050 will be more suitable for species expansion compared to the current one. Unfortunately, the AbR has limited gene flow, since the seeds fall close to the parent plant and are dispersed by rain and gravity to nearby areas, germinating relatively close to the mother plant [14,36]. The absence of long dispersal mechanisms will be challenging for the species expansion, despite the existence of predicted suitable climatic conditions in the future. Climate change, by itself, will not affect the species’ future distribution, at least for the considered period and scenario. The species’ conservation will depend on its dispersal capacity, but also on the extent of land use changes.

Considering the LULC changes from 1951–1980 to 2018, the species’ current distribution was mainly due to past LULC changes. The principal changes in LULC were the conversion of Agriculture and Forestry areas. This study revealed that the land use changes through the expansion of Agriculture (into Orchards) and Forest decline (from Chestnuts and Oaks), caused a direct loss of suitable habitat for the species [15,28]. Thus, Orchard expansion with the consequent increase in herbicide and fertilizer application, and habitat fragmentation have contributed to the decline, in quantity and quality, of the AbR natural habitat, besides general biodiversity. Indeed, large-scale evidence of N accumulation in the root zones of agricultural soils, due to this chemical input, is expected [39], and cherry orchards are generally managed in the region by using fertilization with nitrogen. The impact of land use was also verified in a taxon with a restricted habitat in Portugal, *Cistus ladanifer* subsp. *sulcatus*, together with global warming [40], due to future possible fragmentation and the impossibility of the species’ migration in the future. Indeed, human activities in species fragmentation and constraint are quite common, in particular in the Mediterranean region, such as in the case of *Abies nebrodensis* in Sicily [41]. Additionally, the occurrence and increase in forest fires promoted the expansion of invasive species, namely *Acacia dealbata*, decreasing the species’ habitat.

Endemic species are generally more vulnerable to human activities and habitat changes, with a consequent higher risk of extinction [42]. AbR’s natural habitat is located in a place with high human impact, either through agricultural and forestry exploration or proximity to urban areas, provoking habitat changes and losses. The conservation of an endemic and endangered species is a challenge, and the conservation of AbR is no exception, although public awareness of species preservation exists to a certain extent. Therefore, the appropriate management of AbR areas is vital for successful conservation and further biodiversity maintenance. Furthermore, the species’ medicinal and other economic properties [24] emphasize further the importance of conservation, including a putative increase in area due to potential economic species use. Nevertheless, studies about the impact of soil N accumulation in AbR are unknown, which might be an extra driver to take into consideration in this taxon’s protection.

To allow AbR in situ conservation by creating a seed bank, the factors that affect annual seed production ought to be studied: the physiological and morpho-anatomical characteristics and habitat-related factors, such as edaphoclimatic factors, herbivores, and pollinators’ behavior [43].

Based on the species knowledge and the results presented in this study, the following management guidelines are proposed for habitat maintenance and species conservation: (1) alert people about the need for the species’ conservation; (2) develop sustainable cherry orchard management; (3) provide training and incentives for landowners to use agricultural and forestry practices compatible with conservation, including selective cleaning of cherry slopes after AbR flowering/fruiting (late spring/early summer), minimizing orchard terrace mobilization, reducing the use of herbicides and fertilizers, and reducing forest density; (4) promote the conservation of the native species, namely *Quercus pyrenaica* and *Castanea sativa*; (5) identify suitable areas for species transplantation, since vegetative reproduction will help to increase the number of individuals, despite decreasing genetic diversity [14]; (6) conserve AbR germplasm (including cryopreservation, tissue culture, seed maintenance at low temperatures) for future restoration and regeneration initiatives and rehabilitation of areas with reduced number of individuals; (7) produce seed through controlled crosses with plants located far away from each other, to increase genetic diversity and decrease biparental inbreeding; and (8) to check the impact of soil N accumulation on the taxon.

## 4. Materials and Methods

### 4.1. Study Area

**Figure 8:**
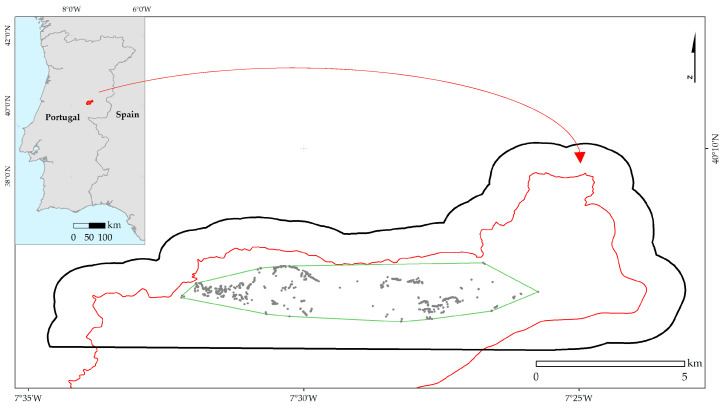
Location of the study area (SA—black line). Serra da Gardunha Regional Protected Landscape (red line). Spatial distribution of AbR occurrences (Gray dots) and the corresponding occurrence area (OA—green line).

The study area, with a latitude between 40.11° N and 40.17° N and a longitude between 7.58° W and 7.38° E, was located on the northern side of the Serra da Gardunha Regional Protected Landscape (Figure 8) [44]. The Serra da Gardunha is included on the national SIC list of the Natura 2000 Network and is also considered a Conservation Special Zone (ZEC) under the Habitats Directive (92/43/EEC). AbR occurs preferably in the core areas of their habitat designated as Sites of Community Importance (SCIs)—regional protected areas under the Council Directive 92/43/EEC of 21 May 1992, on the conservation of natural habitats and on wild fauna and flora and, which are included in the list of natural and semi-natural habitats: Site Code Gardunha: PTCON0028 Annex B-I of Dec. Law No. 49/2005.

### 4.2. Species Occurrence Data

The presence dataset was obtained from two datasets: (1) 90 occurrences, resulting from the fieldwork conducted in 2005 by Esteves [36]; and (2) 786 occurrences, recorded in the fieldwork conducted in 2021 by the CULTIVAR project team. All 786 records (Figure 8) were referenced to the WGS84 coordinate system and used throughout this work. The final dataset was reduced to one occurrence per unit cell (~100 m) to attenuate spatial autocorrelation, resulting in 201 presence records used in the species modeling.

### 4.3. Environmental Data

**Figure 9:**
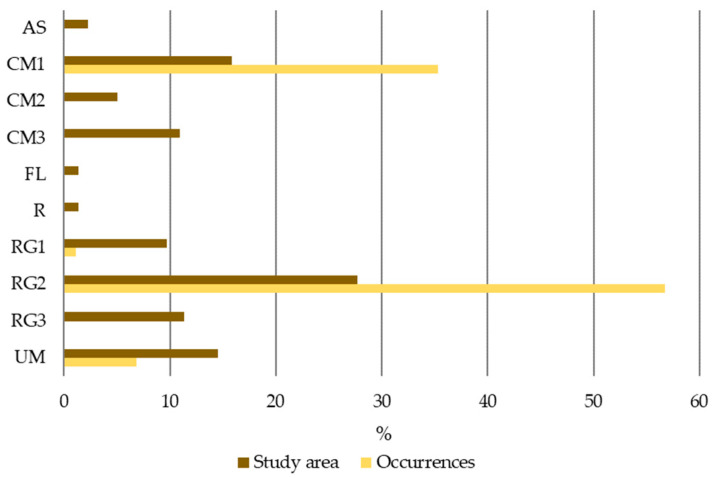
The soil type in the study area and species occurrences. The soil type description and codes as in Table A1.

The SDM was constructed using occurrence data and environmental variables. Three sets of variables were considered: bioclimatic (temperature- and precipitation-related variables), topographic (elevation, slope, and aspect), and soil. The 19 bioclimatic variables for 1970–2000, with 30 s (~1 km^2^) spatial resolution, were downloaded from WorldClim v2.1 [45] (Table 4). The future climate projection for 2050 was also extracted from WorldClim v2.1, considering the Coupled Model Intercomparison Project 6 (CMIP6). The EC-Earth3-Veg GCM (2041–2060) [46] and the Shared Socioeconomic Pathways (SSPs) SSP3-7 were considered. The soil data were extracted from Portuguese Soil Cartography at a scale of 1:100,000 [47]. Ten soil classes were used (Figure 9, Table A1).

AbR is an endemic species with a small range size that required a higher resolution of the environmental variables. The environmental layers were resampled to the same resolution to overcome this issue. Therefore, the bioclimatic and soil variables were downscaled, and the digital elevation model (DEM) was upscaled to 100 m cell size. The DEM was used to derive three surface variables: elevation, slope, and aspect.

Some of these variables are highly correlated, and thus appropriate reduction is crucial to obtain more reliable results and improve the model’s prediction accuracy [48,49]. To eliminate the collinearity among the 23 environmental variables, the R package *virtualspecies* v1.5.1 [50] was used. The *removeCollinearity* function was applied to check the variables’ collinearity using Pearson’s correlation coefficient [51]. The multicollinearity cutoff = 0.8 was applied to select the noncollinear variable subset. The final set included seven variables. Afterwards, the percent contribution, the permutation importance, and the Jackknife test of variable importance were employed to select the most important variables (See Section 4.5).

### 4.4. Land Use and Land Cover Data

The Portuguese land use and land cover (LULC) maps were downloaded from the National Geographic Information System (SNIG—https://snig.dgterritorio.gov.pt/ (accessed on 24 February 2023)) of the Directorate-General for the Territory (DGT—https://www.dgterritorio.gov.pt/ (accessed on 24 February 2023). For the study period 1951–1980, the Agriculture and Forestry Map of Mainland Portugal (MAF1951–1980), the oldest source of Portuguese agricultural and forestry cartography, was used [52]. Between 1995 and 2018, land use and land cover maps (COS) were considered: COS1995, COS2007, and COS2018 [53]. The COS maps were obtained from orthophoto maps photointerpretation and have a minimum mapping unit (MMU) of 1 ha and a 1:25,000 scale. The COS nomenclature follows a hierarchical system of LULC classes, which can be consulted in [54]. In 2022, the DGT has conducted a correspondence between the MAF1951–1980 and COS2018 nomenclatures to make MAF1951–1980 compatible with the nomenclature of the COS series.

### 4.5. Modeling

The SDMs relate species occurrences to environmental variables to define ecological niches and predict species potential distributions. The Maximum Entropy Species Distribution Modelling, version 3.4.1 [55] (MaxEnt),was used to model and project the AbR current distribution into the future, 2050. This machine learning software estimates the geographic distribution of a species by finding the most uniform distribution subject to constraints derived from environmental conditions at the species’ observed areas of occurrence [56] and became the first choice when presence-only data were available, which was the case in this study. MaxEnt’s default output Cloglog was considered, which provides an estimate between 0 and 1 of the probability of presence [57], with “0” corresponding to an unsuitable location and “1” to a suitable location for AbR. The MaxEnt parameter settings also included a maximum number of 2000 iterations to enable model convergence, and 2000 points were randomly selected from the study area as background. The remaining parameters, such as regularization multiplier (1), prevalence (0.5), convergence threshold (0.00001) and features, were set to default settings. The seven variables previously selected were further explored using the MaxEnt approaches: the percent contribution, the permutation importance, and the Jackknife test results. The area under the curve (AUC) of the receiving operator characteristics (ROC) was used to assess the model’s accuracy. The AUC values range from 0 to 1, with higher values corresponding to better model prediction [58].

The current and future species potential distributions were analyzed using the MaxEnt output maps. These maps were reclassified into five suitability classes using an equal interval approach: (1) non-suitable area [0–0.2[, (2) low-suitability area [0.2–0.4[, (3) regular-suitability area [0.4–0.6[, (4) medium-suitability area [0.6–0.8[, and (5) high-suitability area [0.8–1.0]. The species’ suitable and unsuitable habitats were assessed through binary presence-absence maps obtained by applying the 10th percentile training Cloglog presence threshold [59]. The values under the threshold were set to 0, representing unsuitable areas, and the values over the threshold were set to 1, representing suitable areas. The MaxEnt output analysis was carried out in ArcGIS software, version 10.8.1.

### 4.6. Land Use and Land Cover (LULC) Change Analysis

To assess LULC changes over time (1951–1980, 1995, 2007, and 2018), both the study area and the species occurrence area were considered (Figure 8). The LULC change analysis for the study area was performed considering eight major classes of the first hierarchical level: (1) Artificialized territories, (2) Agriculture, (3) Grasslands, (4) Agroforestry areas, (5) Forests, (6) Shrublands, (7) Sparsely vegetated areas, and (8) Water bodies (SA, Table A2).

The species’ occurrence area was defined considering the AbR occurrences and using the "Minimum Bounding Geometry" tool and the "Convex hull" geometry type of ArcGIS software. For the occurrence area, 14 classes were considered: four classes of the first level (Artificialized territories, Agriculture (Agriculture excluding permanent crops), Grasslands, and Shrublands), and 10 classes of the fourth level (Vineyards, Fruit orchards, Olive groves, Cork oak, Other oaks, Chestnut, Eucalyptus, Invasive species, Deciduous forests, and Pine woods) (OA, Table A2).

The LULC change analysis was carried out in ArcGIS software, version 10.8.1.

## 5. Conclusions

AbR is an endemic plant that only exists in a narrow area of the Serra da Gardunha Regional Protected Landscape, located in mainland Portugal. Considering its endangered status and valuable medicinal properties, its conservation and increase in area, reducing the risk of extinction is of utmost importance. The main constraints to its conservation are its small occurrence area and the low numbers of individuals remaining. The increase in the area of cherry orchards and the expansion of invasive species like *Acacia dealbata* and of urban areas contribute to the reduction of suitable habitat for the species.

The SDM MaxEnt was used to model AbR’s current and future distribution. The results suggest that future climatic conditions (2050) will be favorable for the species’ development. Considering the LULC analysis between 1951–1980 and 2018, it was concluded that the observed changes in land use contributed to the decrease in suitable habitat for AbR. Assuming future changes in LULC, the area of cherry orchards will continue to increase due to cherry’s economic importance for the region, and the species’ occurrence area will be reduced, which will be more pronounced if conservation measures are not implemented.

The current study has highlighted the importance of assessing climate change together with LULC changes. Thus, it is recommended that in future studies, the LULC variable be considered in the species distribution modeling to quantify which of the two direct drivers, climate and land use change, will be more relevant for the future distribution of the species, to provide decision-makers with the necessary information to adopt preservation and conservation measures to prevent the species’ extinction. In any case, conservation measures should be taken into consideration due to the species’ current conservation status, together with habitat management measures due to orchard expansion, herbicide use, declines in oak and chestnut areas, the probability of an increase in wildfires, the difficult control of invasive species, and the putative influence of N accumulation, which means the promotion of agricultural and forestry practices compatible with the species’ conservation and habitat restoration.

## Figures and Tables

**Table 1:** Percent contribution, permutation importance, and regularized training gain for the environmental variables included in the model.

Variable	Percent Contribution	Permutation Importance	Training Gain without	Training Gain with Only
BIO4	68.07	59.69	1.06	0.77
BIO7	9.83	11.20	1.18	0.10
Aspect	7.20	1.84	1.15	0.28
Soils	5.90	0.00	1.19	0.43
BIO2	4.96	22.46	1.16	0.44
BIO15	2.76	4.08	1.18	0.35
BIO12	1.28	0.73	1.19	0.53

**Table 2:** The LULC changes from 1951–1980 to 2018 (%) for the study area.

LULC Class	MAF1951–1980	COS1995	COS2007	COS2018
Artificialized territories	2.0	4.5	5.2	5.3
Agriculture	46.8	44.3	43.6	44.5
Grasslands	0.0	0.7	1.1	0.8
Agroforestry areas	0.4	0.6	0.9	0.9
Forests	39.7	43.7	44.5	43.8
Shrublands	11.1	6.1	4.6	4.6
Sparsely vegetated areas	0.0	0.0	0.0	0.0
Water bodies	0.0	0.1	0.1	0.1

**Table 3:** The LULC changes from 1951–1980 to 2018 (%) for the species occurrence area.

LULC Class	MAF1951–1980	COS1995	COS2007	COS2018
Artificialized territories	1.0	2.9	3.5	3.4
Agriculture	13.7	10.5	7.0	6.8
Agriculture–Vineyards	3.6	0.1	0.1	0.1
Agriculture–Fruit orchards	15.0	44.8	48.5	49.0
Agriculture–Olive groves	4.8	1.1	1.3	1.3
Grasslands	0.0	0.1	0.3	0.2
Forests–Cork oak	0.1	0.0	0.0	0.0
Forests–Other oaks	0.7	6.8	6.6	6.3
Forests–Chestnut	21.5	4.5	4.8	4.6
Forests–Eucalyptus	2.1	0.2	0.2	0.2
Forests–Invasive species	0.0	0.0	2.9	2.9
Forests–Deciduous forests	0.4	5.5	1.8	1.8
Forests–Pine woods	30.8	19.5	20.2	19.8
Shrublands	6.3	4.0	2.8	3.6

**Table 4:** Summary statistics for the current environmental variables for the study area and species occurrences.

Symbol	Variable	Study Area				Species Occurrences			
		Min	Max	Mean	Std	Min	Max	Mean	Std
BIO1	Annual mean temperature (°C)	12.0	14.7	14.0	0.5	12.9	14.3	13.7	0.3
BIO2	Mean diurnal range (°C)	8.0	10.1	9.5	0.4	8.7	9.7	9.3	0.2
BIO3	Isothermality (%)	33.3	40.0	37.7	1.3	35.2	38.5	36.9	0.6
BIO4	Temperature seasonality (%)	545.5	582.9	564.3	8.8	561.1	581.0	573.6	3.6
BIO5	Max. temperature of the warmest month (°C)	26.2	29.4	28.6	0.5	27.5	29.0	28.4	0.3
BIO6	Min. temperature of the coldest month (°C)	2.3	4.0	3.5	0.3	2.9	3.7	3.3	0.1
BIO7	Temperature annual range (°C)	23.8	25.6	25.1	0.3	24.6	25.4	25.1	0.2
BIO8	Mean temperature of the wettest quarter (°C)	6.4	9.1	8.3	0.5	7.2	8.6	7.9	0.3
BIO9	Mean temperature of the driest quarter (°C)	19.6	21.8	21.3	0.4	20.5	21.5	21.2	0.2
BIO10	Mean temperature of the warmest quarter (°C)	19.7	21.8	21.3	0.3	20.5	21.5	21.2	0.2
BIO11	Mean temperature of the coldest quarter (°C)	5.6	8.4	7.6	0.5	6.5	7.9	7.3	0.3
BIO12	Annual precipitation (mm)	977.2	1263.2	1058.9	51.8	1019.7	1176.5	1103.4	32.5
BIO13	Precipitation of the wettest month (mm)	145.2	197.5	159.7	8.5	153.9	179.9	166.1	5.2
BIO14	Precipitation of the driest month (mm)	9.0	13.7	10.9	1.0	10.0	13.0	12.0	0.6
BIO15	Precipitation seasonality (%)	57.8	59.2	58.4	0.2	57.9	58.7	58.2	0.1
BIO16	Precipitation of the wettest quarter (mm)	408.2	540.0	446.9	23.0	430.1	499.5	466.3	13.6
BIO17	Precipitation of the driest quarter (mm)	52.0	80.5	59.6	5.0	55.2	71.6	64.0	3.2
BIO18	Precipitation of the warmest quarter (mm)	52.2	81.8	60.9	5.5	55.2	73.6	64.3	3.4
BIO19	Precipitation of the coldest quarter (mm)	397.2	522.6	437.9	22.4	421.8	487.7	457.2	13.7
E	Elevation (m)	425.1	1133.1	585.5	119.2	505.4	848.6	669.6	62.7
S	Slope (%)	0.3	57.5	15.9	10.9	5.0	45.2	27.6	8.3
A	Aspect (°)	0.0	360.0	188.3	121.4	0.1	359.6	219.5	151.1

Legend: Min—minimum; Max—maximum; Std—standard deviation.

## Data Availability

Data used in this study can be made available by contacting alicemalmeida@ipcb.pt.

## Appendix A

**Table A1:** Soil type descriptions. The soil types in the study area and in AbR occurrences.

Code	Classification	Description	Study Area (%)	Occurrences (%)
AS	AS	Social Area	2.3	0.0
CM1	CM.dy.cr(ha)	Chromi-Dystric Cambisols	15.8	35.3
CM2	CM.dy.ha	Hapli-Dystric Cambisols	5.0	0.0
CM3	CM.dy.ha(cr)	Hapli-Dystric Cambisols	10.9	0.0
FL	FL.dy	Distric Fluvisols	1.3	0.0
R	R	Rocky Outcrops	1.4	0.0
RG1	RG.ai.dy	Dystri-Aric Regosols	9.7	1.1
RG2	RG.lep.dy	Dystri-Epileptic Regosols	27.7	56.8
RG3	RG.lep.dy(sk)	Dystri-Epileptic Regosols	11.4	0.0
UM	UM.lep.hu	Humic Epileptic Umbrisols	14.5	6.8

**Table A2:** Descriptions of land use and land cover (LULC) classes for the study area (SA) and occurrence area (OA).

Area	LULC Classes	Description
SA/OA	Artificialized Territories	Urban fabric; industrial, commercial, and transport units; mine, dump, and construction sites; artificial non-agricultural vegetated areas.
SA	Agriculture	Arable land, lands under a rotation system used for annually harvested plants and fallow lands, which are rain-fed or irrigated and for permanent crops. Areas of annual crops associated with permanent crops on the same parcel, annual crops cultivated under trees, areas of annual crops, meadows and/or crops and pastures mixed with natural vegetation.
OA	Agriculture	Agriculture (SA) excluding permanent crops.
	Vineyards	Permanent crops, lands not under a rotation system, with vineyards.
	Fruit orchards	Permanent crops, lands not under a rotation system, with fruit orchards, trees or shrubs of one or more species intended for fruit production.
	Olive groves	Permanent croplands not under a rotation system, with olive groves.
SA/OA	Grasslands	Pasture lands that are permanently used (at least 5 years) for fodder production. Includes natural or sown herbaceous species, unimproved or lightly improved meadows and grazed or mechanically harvested meadows.
SA	Agroforestry areas	Landscapes in which crops and pastures are intimately mixed with Mediterranean forest (*Quercus suber*, *Quercus rotundifolia* and other oaks) and natural vegetation or natural areas.
	Forests	All forest species resulting from natural regeneration, seeding or planting.
OA	Cork oak	*Quercus suber* forest.
	Other oaks	*Quercus pyrenaica*, *Quercus robur*, *Quercus faginea* forests, and other oaks but not including *Quercus suber* and *Quercus rotundifolia*.
	Chestnut	*Castanea sativa* forest.
	Eucalyptus	*Eucalyptus* spp. forest.
	Invasive species	Invasive forest (e.g., *Acacia dealbata*, *Ailanthus altissima*).
	Deciduous forests	Broadleaved forests (e.g., *Salix* spp., *Populus* spp., *Platanus* spp., *Alnus glutinosa*, and *Juglans regia*) for wood purposes.
	Pine woods	*Pinus pinaster* or *Pinus pinaster* and *Pinus pinea* mixed stands.
SA/OA	Shrublands	Natural areas of spontaneous vegetation, sparsely with shrubs or very dense.
SA/OA	Sparsely vegetated areas	Natural areas with little or no vegetation, including recently burnt areas and bare rock.
SA	Water bodies	Inland wetlands.
